# The role of psychological factors in predicting latrine ownership and consistent latrine use in rural Ethiopia: a cross-sectional study

**DOI:** 10.1186/s12889-018-5143-0

**Published:** 2018-02-08

**Authors:** Fikralem Alemu, Abera Kumie, Girmay Medhin, Janvier Gasana

**Affiliations:** 1 Ethiopian Institute of Water Resources, Water and Health Program, Addis Ababa University, Addis Ababa, Ethiopia; 2 School of Public Health, College of Health Sciences, Addis Ababa University, Addis Ababa, Ethiopia; 3 Aklilu Lemma Institute of Pathobiology, Addis Ababa University, Addis Ababa, Ethiopia; 4 Kuwait University, Faculty of Public Health, Department of Environmental & Occupational Health, Safat 13110, Kuwait, Kuwait

**Keywords:** Latrine ownership, Consistent latrine use, Rural, Ethiopia, Psychological factors

## Abstract

**Background:**

Inadequate sanitation is one of the leading causes of disease in poor and middle-income countries.

**Objective:**

The objective of the study was to identify the psychological factors that predict latrine ownership and consistent latrine use in the rural Becho district of central Ethiopia.

**Method:**

A quantitative, cross-sectional, community based study was conducted. A total of 1047 heads of household were interviewed using a structured questionnaire. Ownership of latrine and consistent latrine use constituted the outcome variable of the study. Data were entered using Epi Info version 3.5.4 and were analyzed using SPSS version 20.

**Results:**

Of the 1047 households, 73% owned a traditional pit latrine. Among the psychological factors, attitude (AOR 1.70; 95% CI 1.21–2.37) and injunctive norm (AOR 6.18; 95% CI 4.46–10.44) were positively and significantly associated with latrine ownership. Among the demographic factors**,** having a family size of more than six (AOR = 1.43; 95% CI 1.01–1.97, having a child attending school (AOR = 1.88; 95% CI 1.17–3.02), and having a high school education (AOR = 1.98; 95% CI 1.34–2.87) were significantly associated with latrine ownership. With respect to exposure to communication about sanitation (the cues to action), households that had a family member who took part in Community Led Total Sanitation and Hygiene (CLTSH) triggering were three times more likely to be latrine owners than those who did not participate in CLTSH triggering (95% CI 1.92–4.78.) Results from adjusted logistic regression analysis of potential predictors of consistent latrine use showed that having a positive attitude (AOR 7.00; 95% CI 4.55–10.55), owning of a latrine that had superstructure (AOR 2.3 95% CI 1.47–3.48), having a clean latrine (AOR 1.69 95% CI 1.00–3.00), and having a latrine with a protected door (AOR 1.94; 95% CI 1.10–3.48) were significantly associated with consistent latrine use.

**Conclusion:**

The study findings showed that attitude and injunctive norm are the psychological predictors of latrine ownership, and consistent latrine use was associated with attitude, cleanliness of the latrine, and its privacy. Hence, sanitation intervention needs to focus on changing societal norms, attitudes, and the promotion of latrine quality.

## Background

Improvement in sanitation has a profound effect on human health (1–6). Evidence suggests that improved sanitation reduces diarrheal disease by up to 36% and intestinal parasitic infection by up to 50% [1–5]. However, in 2015, an estimated 2.4 billion people still lacked access to improved facilities [6]. More than 1.8 million deaths occur globally each year as the result of diarrheal disease, and children under age 5 accounted for 90% of those deaths [7–9].

Ethiopia has a high level of diseases due to poor sanitation [5, 10]. Diarrhea is the second-leading cause of death in children under age five [11]. More than 26 million Ethiopians are infected with intestinal parasites [12], and approximately 38% of children under age five are stunted [13]. Every year, an estimated 64,540 child deaths occur in Ethiopia due to inadequate water, sanitation, and hygiene [14]. Ethiopia launched a health extension program a decade ago. Health extension workers provide door-to-door health education to the households [15, 16]. In 2011, Ethiopia adopted the Community Lead Total Sanitation and Hygiene (CLTSH) programs as a national sanitation strategy [17]. Although these programs have been successful in decreasing open defecation [18], there was no progress in access to improved sanitation in Ethiopia, increasing from a baseline of 3% in 1990 to 28% in 2017 [6].

Lessons learned from the implementation of sanitation programs in Sub-Saharan Africa and elsewhere during the past two decades show that programs that provide or subsidize a toilet without addressing behavioral changes result in unused sanitation facilities, and the facilities themselves are often not maintained [19–22]. An increasing body of literature suggests that the effectiveness of sanitation depends not only on the provision of sanitation facilities but also, and most importantly, on the compliance of individuals [23–25]. Models of behavioral change suggest that behavioral change interventions should be planned based on the identified factors of the targets [26]. Although there is growing interest and focus on sanitation behavior by program planners and practitioners, there are relatively few studies on sanitation behavior that can inform the design of successful programs and strategies [27]. Limited studies applied theoretical models and frameworks to understand factors that determine the adoption and consistent use of sanitation facilities in developing countries [28]. However, most behavioral change programs on sanitation focus on educating people about risks of diseases and infections without sufficient grounding in behavioral theory [22].

The Risk, Attitude, Norm, Ability, Self-regulation (RANAS) model underlines the importance of psychological factors in predicting Water, Hygiene and Sanitation (WASH) behaviors. The model proposes specific intervention strategies for specific factors [27]. Here, we present a study guided by the RANAS model to investigate the individual psychological predictors of latrine adoption in the rural Becho district of Ethiopia. The Becho district was purposely selected because sustaining latrine adoption has been a challenge in the area [29]. The study answers the following research question: What are the psychological determinants of latrine ownership and consistent latrine use in the rural Becho district of Ethiopia?

## Methods

### Study design

A community-based cross-sectional quantitative study was employed

### Study area and study population

Ethiopia has nine regional states and two city administrations. Each region is subdivided into a set of zones, and each zone is divided into woreda (equivalent to a district). A kebele is the lowest-level administrative structure. This study was conducted in the Oromia region, a rural kebele of the Becho woreda from May 1 to May 30, 2015. Becho is located 80 km from Addis Ababa, the capital city of Ethiopia. Based on the 2007 national demographic census, the district had a projected total population of 88,550 in 2015, 80.4% of which were rural residents [30]. According to the 2014 woreda health report, the percentage of the population owning any kind of latrine in the Becho district was 57% [29].

### Study participants

Study participants included heads of household who had been residents of the Becho district for at least six months prior to the study period. Those temporarily staying in the study area for different purposes, such as caring for the household, were excluded from the study.

### Sample size and sampling procedure

A sample size of 1146 was estimated using the software Epi Info Version 3.5.4 (Centers for Disease Control and Prevention, Atlanta, GA, USA), considering 68% latrine coverage (i.e., any kind of latrine) from Ethiopia’s 2014 mini DHS report [31]; the design effect was 2, the margin of error was 0.04, and the confidence level was 95%. The Becho district has a total of 19 kebeles, and 17 are rural. We implemented a two-stage sampling technique. First, we selected eight rural kebeles (clusters) using simple random sampling, and then study participants from each kebele were selected using a systematic random sampling method. Using a sampling interval of six, the first household in each kebele was selected by simple random sampling, and then data collectors enrolled every sixth household to participate in the study.

### Data collection method and procedure

Data were collected using a structured questionnaire that was originally prepared in English and then translated into the local language (Oromiffa). The questionnaire was pretested among 50 households residing in a non-sampled cluster district, and the modified questionnaire was used in the final survey. We assessed the challenges of social desirability during pretesting, and some of the language in the questionnaire was modified. In addition, the data collectors were recruited from the study area to minimize the unknown urban person effect. Three supervisors with a master’s degree and ten data collectors with at least a diploma-level education who speak the local language were recruited to administer the survey. Training was provided to data collectors and supervisors over three days. During the data collection, the first author and supervisors closely examined the data collectors administering the interview. All completed questionnaires were checked by the supervisors in the field for missing values, skip patterns, and logic. Corrections for possible inconsistencies were made in the field. For incomplete data, the respondents were re-visited the next day. Supervisors randomly selected 25% of the completed questionnaires from each data collector and rechecked by visiting the households.

### Variables and measurements

#### Outcome variable

Latrine ownership and consistent latrine use were the outcome variables for investigating the predicting factors. Latrine ownership was measured by asking the participants if they own any kind of latrine, which was confirmed through observation. The frequency of latrine use over the last week by latrine owners was assessed using a 4-point Likert scale of ‘always’, ‘very often’, ‘less often’, and ‘never’. Respondents who reported always using a latrine were categorized as consistent latrine users, and the rest were categorized as inconsistent latrine users.

#### Predictor variables

Psychological factors, demographic factors, and cues to action were investigated as predictors of latrine ownership and consistent latrine use. The items in the questionnaire that assessed psychological factors are displayed in Table 1. Psychological factors included perceived risk (perceived vulnerability and perceived severity), attitude, and perceived norm (both injunctive and descriptive norms) [27]. We assessed the influence of latrine quality on consistent latrine use, which included cleanliness and having a superstructure or door that could provide privacy [32]. Questions for the psychological variable were adopted from prior studies that examined psychological predictor of a specific behavior [33–35], and RANAS model [36, 37] and we contextualized it for latrine ownership and consistent latrine use. Overall, the questions used to measure psychological variables were developed using the theory of planned behavior (TPB)/theory of reasoned action (TRA) [38, 39] and the Health Belief Model (HBM) [32]. These theories have been widely used in the context of sanitation and hygiene behavior [38, 39]. The participants’ intention to build a latrine and their self-efficacy were also measured and reported.

**Table 1:** Summary of measurements used to assess the psychological predictors of latrine ownership and consistent latrine use

Factor	Items	Responses	values
Outcome variables			
Latrine ownership	Ownership of latrine	No/Yes for ownership of latrine	0/1
Consistent latrine use			
Predictor variables			
Risk Perception			
Vulnerability	How high or low are the chances that you contract diarrheal disease when defecating in the open field?	Five point scale, that ranges from almost Very low, to Very high	1 to 5
Severity	If you have diarrheal disease because of open defecation, how severely would that impact your life?	Five point scale, that ranges from almost Very low, to Very high	1 to 5
Attitude (affective)			
	How much beneficial/important it is building Five point scale, that ranges from almost Very low, to Very high your own latrine in the one year		1 to 5
	How much beneficial/important it is to defecate using latrine regularly	Five point scale, that ranges from almost Very low, to Very high	1 to 5
	How much do you like to use latrine?	Five point scale, that ranges from almost Very low, to Very high	1 to 5
	How much do you do you enjoy defecating in latrine?	Five point scale, that ranges from almost Very low, to Very high	1 to 5
Self-efficacy (latrine ownership) Injective norm (latrine ownership)	How much ability you think you have building your own latrine in the next one year	Five point scale, that ranges from almost Very low, to Very high	1 to 5
	Most of the people in my village think I should have my own latrine	Five point scale, that ranges from completely disagree to completely agree	1 to 5
	People in my village will judge me if I don’t have my own	Five point scale, that ranges from completely disagree to completely agree	1 to 5
Descriptive norm (latrine use)	Most of the people I know in the community defecate using latrine regularly	Five point scale, that ranges from completely disagree to completely agree	1 to 5
	How many of your neighbors use latrine for defecation?	Five point scale, that ranges from almost nobody to almost all	1 to 5
	Using latrine regularly is the right thing to do because everybody does so	Five point scale, that ranges from completely disagree to completely agree	1 to 5
Injunctive norm (latrine use)	people who are important to me approve /disapprove that you use latrine	Five point scale, that ranges from completely disagree to completely agree	1 to 5
	Defecating using latrine is regularly is something that most of the people in my village think	Five point scale, that ranges from completely disagree to completely agree	1 to 5
	People in my village will judge me if I defecate in the open field	Five point scale, that ranges from completely disagree to completely agree	1 to 5
Cues to action			
Exposure to Health education (HEW)		Yes/No	
Exposure to CLTSH triggering		Yes/No	

Items and scales used to measure the psychological factors for predicting latrine ownership and consistent latrine use

##### Risk perception

As described by the Health Belief Mode (HBM), we measured perceived vulnerability, which is the extent to which the person believes that she/he is susceptible to diarrheal disease due to contamination from open defecation; and perceived severity, which is the perception of the severity of the illness consequences [32]. A Likert scale (1–5) was adopted to measure perceived vulnerability and perceived severity. Responses of 1–3 were recorded as low vulnerability and severity, whereas a response of 4 or 5 was recorded as high vulnerability or severity.

##### Norm perception:

The effects of social norm perception on a particular behavior is described by the theory of normative social behavior (TNSB) [40], which is analogous to the subjective norm in the theory of reasoned action (TRA) [38]. Social norm is differentiated as the descriptive norm—an individual’s perception about how others behave—and an injunctive norm, which is the perception about how others expect them to behave [41, 42]. In this study, the descriptive norm for latrine use is defined as the participant’s perception about the prevalence of latrine use. It was measured using 3 items that were adopted from studies on social norms [43]. The composite score, ranging from 4 to 15, was then recorded with high descriptive norm latrine use being a score greater than 10.6, and low descriptive norm for a score less than or equal to 10.5. The injunctive norm is the participant’s perception that important referents expect them to behave. The injunctive norm for latrine ownership was measured using two items. The composite score, ranging from 2 to 10, was recorded as high injunctive norm latrine ownership for a score of 8 to 10, and low injunctive norm form latrine ownership for a score of 2 to 7. The injunctive norm for consistent latrine use was measured using 3 items. The total score of three items ranged from 3 to 15, which was recorded as high and low for scores of 3 to 10 and scores greater than 10, respectively.

##### Attitude:

The TRA/TPB explains that attitude, a feeling arising when performing or thinking about a behavior, predicts a certain behavior [39, 44]. We assessed attitude by asking three questions about the participants’ feelings regarding latrine use. The composite score of the three items ranged from 4 to 15, which was recorded as high attitude, for a score greater than 10, and low attitude, for a score 4 to 10.

##### Perceived ability: (self-efficacy)

The theory of planned behavior describes the individual’s perception about their capacity to perform the desired behavior as self-efficacy. Self-efficacy in the ability to build a latrine in the next 12 months was assessed for individuals who did not own a latrine using a Likert scale.

##### Cues to action:

According to HBM, cues to action are strategies to activate the readiness of individuals, such as providing information, promote awareness, or reminders. Participants were asked about their participation in any sanitation promotion programs or their exposure to interpersonal and mass media communication on sanitation. Responses were recorded as reported by the participants.

##### Behavioral intent (intention):

TRA/TPB propose that a person’s intention when combined with perceived behavioral control will help predict behavior [38]. In this study, intention was defined as the behavioral intention to build a latrine in the coming year and was assessed only in those who did not own a latrine. The score, ranging from 1 to 5, was recorded as high for the scores of 4 to 5 and low for the scores of 1 to 3.

##### Latrine quality:

Latrine quality was measured by observation and the subjective judgement of the data collectors. The cleanliness of a latrine was good if there was no sign of excrement anywhere and if the latrine was clean and tidy, fair when some excrement was found only around the pan/squatting plate, and poor when there was excrement on the floor or walls. Good and fair levels of cleanliness were categorized as clean, whereas poor cleanliness was recorded as unclean. We measured the variable ‘Latrine has a protected entry door’ as Yes when the door was made of a metal sheet, a sheet made of bamboo matting, wood, curved entrance with plastered walls, flat wood, and sheets made of bamboo, and No when there was no door or if there was a cloth curtain, curved entrance, unplastered wall, or plastic sack. The response for “Latrine has superstructure” was “Yes” when the wall was made of brick and cement, metal sheets, stone, stone/mud, sheets of bamboo matting, wood, etc., and the response was “No” when the wall was made of sesame stalk or leaves or if there was no wall.

#### Data management and analysis

Data were entered twice using the software Epi info version 3.5.4. Then, data screening and cleaning was performed by running frequencies and cross-tabulations. The clean data was exported to SPSS version 20 for analysis. The normal distribution assumption for the selected socio-demographic and main independent variables was analyzed using SPSS. Multicollinearity among independent variables was assed using the Shapiro-Wilk test. Descriptive summaries of the dependent variable, and all the potential predictor variables were obtained by running frequencies. Unadjusted logistic regression analyses were conducted to assess any significant association between each potential predicting factor and the outcome variable. Odds ratios with corresponding 95% confidence interval (CI) were used to quantify the degree of association. All variables having a *p*-value ≤0.05 in the bivariate analysis were considered for multivariable logistic regression analysis. Since our study was limited to one time data collection, we were unable to explore the effect of intention and perceived ability on future behavior among non-owners of latrines. Rather, we presented the two variables as descriptive data.

## Results

### Background characteristics of the study participants

One thousand and forty-seven (1047) heads of household participated in the study with a response rate of 92%. The demographic characteristics are summarized in Table 2. The mean age was 42 years, with a SD of 13.3. The majority were married (88.2%), had no formal education (58.2%) or primary education (34.5%), were farmers (96.4%), belonged to the Oromo ethnic group (99%) and were followers of Orthodox Christian religion (99%).

**Table 2:** Demographic characteristics of study participants in Becho district, Oromiya region of Ethiopia, May 2015 (*n* = 1047)

Characteristics	Number (%) who didn’t own latrine	Number (%) who own latrine
Sex		
Male	166 (58.7)	484 (63.4)
Female	117 (41.3)	280 (36.6)
Marital status		
Married	240 (84.8)	683 (89.4)
Single/Divorced/widowed/separated	43 (15.2)	81(10.6)
Education Status		
No education/informal education	193 (68.2)	416 (54.4)
Primary education	77 (27.2)	284 (37.2)
Secondary education and above	13(4.6)	64 (8.4)
Occupation		
Farmer	276 (97.5)	733 (95.9)
Others	7 (2.5)	31 (4.1)
Age		
< 31	59 (20.8)	112 (14.7)
31–50	147 (51.9)	420 (55)
50+	77(27.2)	232 (30.4)
Family size		
1–6	205 (72.4)	455 (59.6)
7–10	74 (26.1)	271 (35.5)
10+	4 (1.4)	38 (5.0)

#### Latrine ownership, past experience and future intention

Among the study participants, 764 (73.0%) owned latrine. The most common latrine type was unimproved traditional pit latrine (98.5%). Of the 283 non-owners, 85% reported having high intention to build latrine within the coming year, but only 47% of latrine non-owners reported high perceived ability to do it. About 98.6% of latrine non-owners had latrine in previous years and there was some kind of convincing evidence of this for 75.3% of non-owners. The listed reasons for ceasing to use latrine were because latrine had collapsed (62%), or latrine was full (28%).

#### Latrine use

Six hundred three (79%) participants reported to consistently use latrines. The proportion of participants that reported “very often”, “less often” or “never” used latrine during the past one week was 132 (17.3%), 11(1.4%), and 19 (2.5%) respectively.

#### The individual psychological factors

The mean, range, and standard for each individual psychological item are summarized in Table 3. The mean (standard deviation) of composite scores were 8.0 (1.8) for injunctive norm of latrine ownership, 10.4(2.3) for injective norm of latrine use, 10.4 (2.3) for descriptive norm (latrine use), 3.5 (1.3) for perceived ability, 13.6 (1.8) for attitude, 4.6 (0.8) for perceived vulnerability, and 4.5 (0.8) for perceived severity respectively.

**Table 3:** Descriptive statistics of the psychological predictors of latrine ownership and consistent latrine use (*n* = 1047)

Psychological factors	Range (Min, Max)	Mean	SD	Number of Items
Risk Perception				
Vulnerability	4 (1, 5)	4.33	0.79	1
Severity	4 (1, 5)	4.48	0.75	1
Attitude	11 (4, 15)	13.63	1.75	4
Injunctive norm (latrine ownership)	8 (2, 10)	.8.0	1.79	2
Self-efficacy (latrine ownership)	4 (1, 5)	3.45	1.29	1
Descriptive norm (consistent latrine use)	11 (4, 15)	10.37	2.32	3
Injective norm (consistent latrine use)	11 (3, 15)	10.37	2.31	3

Min = minimum; Max: maximum

#### Exposure to communication about sanitation (cues to action)

The majority of participants (93.7%) reported that they were advised or motivated by someone to build a latrine; almost all (98.2%) were advised by the government health extension workers; 21% of the respondents reported that their family member took part in CLTSH triggering events during the previous year.

### Psychological predictors of latrine ownership

Results of the logistic regression showing the crude and adjusted effects of four psychological factors, demographic factors, and cues to action on the odds of latrine ownership are summarized in Table 4. In a univariate analysis, latrine ownership was positively and significantly associated with perceived severity (OR 2.71; 95% CI 1.78–4.15), attitude (OR 2.05; 95% CI 1.55–2.71) and injunctive norm (OR 7.71; 95% CI 1.77–4.15). In the multiple logistic regression analysis that considered selected demographic factors, cues to action, and the psychological factor (i.e., perceived vulnerability, perceived severity, attitude, descriptive norm and injunctive norm), showed that 77.6% of the study participants were correctly classified, and 21.7% (Cox and Snell R-square) to 31.4% (Nagelkerke R-square) of the total variability in the outcome was explained by the model (*p*-value < 0.05), and two psychological factors, namely, attitude (AOR 1.70; 95% CI 1.21–2.37) and injunctive norm (AOR 6.18; 95% CI 4.46–10.44)—were positively and significantly associated with latrine ownership. Among the demographic factors**,** those with a family size of more than 6 compared to small-sized families (AOR = 1.43; 95% CI 1.01–1.97) were more likely to be latrine owners, as were households with a child attending school, compared to those who did not have (AOR = 1.88; 95% CI 1.17–3.02), and the head of household having high school education (AOR = 1.98; 95% CI: 1.34–2.87). With respect to exposure to communication about sanitation (cues to action), households with a family member who took part in CLTSH triggering were 3 times more likely to be latrine owners than those who did not participate in CLTSH triggering (95% CI 1.92–4.78).

**Table 4:** Results from logistic regression assessing predictors of latrine ownership in Becho district. May 2015 (*n* = 1047)

Characteristics	Number (%) who didn’t own latrine	Number (%) who own latrine	COR(95%CI)	AOR(95%CI)
1.Psychological factors				
Perceived vulnerability				
Low	58 (20.5)	81 (10.6)	1.00	1.00
High	225 (79.5)	683 (89.4)	1.15 (0.57–2.32)	1.15 (0.57–2.32)
Perceived severity				
Low	46 (16.3)	51 (6.7)	1.00	1.00
High	237 (83.7)	713 (93.3)	2.71 (1.77–4.15)	1.15 (0.57–2.32)
Attitude				
Low	151 (53.4)	250 (32.7)	1.00	1.00
High	132 (46.4)	514 (67.3)	2.05(1.55–2.71)	1.70 (1.21–2.37)*
Injunctive norm (latrine ownership)				
Low	188 (66.4)	316 (41.6)	1.00	1.00
High	95 (33.6)	444 (58.4)	7.71 (5.70–4.60)	6.18 (4.46–10.44)*
2.Cues to action				
Advised by Health extension worker				
No	17 (6.0)	67 (8.8)	1.00	1.0
Yes	266 (94.0)	697 (91.2)	0.67 (0.38–1.15)	0.78 (0.41–1.46)
Family members participated in CLTSH triggering				
No	256 (90.5)	569 (74.5)	1.00	1.00
Yes	27 (9.5)	195 (25.5)	3.25 (2.12–4.50)	3.02(1.88–4.84)*
3.Socio-demographic variables				
Age group				
< 30	59 (20.8)	112 (14.7)	1.00	1.00
30–49	147 (51.9)	420 (55.0)	1.51 (1.04–2.17)	1.33 (0.87–2.03)
50+	77 (27.2)	232 (30.3)	1.58 (1.05–2.38)	1.66 (0.99–2.80)
Marital status				
Single/divorced/widowed				
Separated	43 (15.2)	81 (10.6)	1.00	1.00
Married	240 (84.8)	683 (89.4)	1.51 (1.02–2.24)	1.43 (0.89–2.28)
Education Status				
No education/informal Education	193 (68.2)	416 (54.4)	1.00	1.00
Primary education(grade 1–8)	77 (27.2)	284 (37.2)	1.71 (1.26–2.32)	1.89 (0.93–3.87)
High school and college	13 (4.6)	64 (8.4)	2.28 (1.23–4.25)	1.98 (1.36–2.87)*
Family size				
< =6	205 (72.4)	455 (59.6)	1.00	1.00
> 6	78 (27.6)	309 (40.4)	1.81 (1.35–2.44)	1.41 (1.01–1.97)*
Presence of a school child				
No	53 (18.7)	82 (10.7)	1.00	1.00
Yes	230 (81.3)	682 (89.3)	1.91 (1.31–2.79)	1.97 (1.27–3.06)*

*Significant at *P* < 0.05

### Predictors of consistent latrine use

Results from logistic regression analysis of consistent latrine use as an outcome variable, and demographic, psychological, and latrine quality factors as predictor variables are displayed in Table 5. The univariate regression analysis of each psychological factor (i.e. perceived vulnerability, perceived severity, attitude, descriptive norm and injunctive norm), and consistent latrine use showed that attitude (OR = 6.48; 95% CI 4.44–9.45), perceived vulnerability (OR = 2.17, 95%CI 1.50–3.14), perceived severity (OR = 2.71; 95% CI 1.77–4.15), and injective norm (OR = 1.34, 95%CI 1.00–1.90) were significantly associated with consistent latrine use (OR = 7.45; 95% CI 4.91–11.30). In the multivariate logistic regression, 78.8% of the study participants were correctly classified, and the model explained between 18.2% (Cox and Snell R-square) and 28.3% (Nagelkerke R-square) of the total variability (*p*-value < 0.05). The results show that three factors related to latrine quality, that were a clean latrine (OR = 1.69; 95% CI 1.00–3.00), a latrine with protected door (OR = 1.94; 95% CI 1.10–3.48), and a latrine with a superstructure (OR = 2.26; CI 1.47–3.48) had a significant positive association with consistent latrine use. Factors not associated with consistent latrine use in the univariate logistic regression were cues to action (being advised by a health extension worker or participating in CLTSH triggering) and demographic variables, such as age, gender, family size, education status, and the presence of a child who attended school.

**Table 5:** Results from logistics regression assessing the association between potential predictors and consistent latrine use in Becho district of Ethiopia. May 2015. (*n* = 764)

Characteristics	Number (%) who did not consistently used latrine	Number (%) who consistently used latrine	COR(95%CI)	AOR(95%CI)
Psychological factors				
Perceived vulnerability				
Low	24 (14.78)	57 (9.5)	1.00	1.00
High	138 (85.2)	545 (90.5)	2.17 (1.50–3.14	2.069 (0.97–4.41)
Perceived severity				
Low	11 (6.8)	40 (6.6)	1.00	1.00
High	151 (93.2)	562 (93.4)	2.71 (1.77–4.15)	0.30 (0.11–0.80)
Attitude				
Low	108 (66.7)	142 (23.6)	1.00	1.00
High	54 (33.3)	460 (76.4)	6.48 (4.44–9.45)	7.45 (4.91–11.30)
Descriptive norm (latrine use)				
High	71 (43.8)	257 (42.8)	1.00	1.00
Low	91 (56.2)	343 (57.2)	1.04 (0.73–1.48)	0.95 (0.63–1.44)
Injunctive norm (latrine use)				
Low	184 (65.0)	405 (53.0)	1.00	1.00
High	99 (35)	359 (47.0)	1.34(1.00–1.90)	1.23 (0.80–1.90)
Demographic variables				
Age group				
< 30	22 (13.6)	90 (15.0)	1.00	1.00
30–49	92 (58.6)	328 (54.5)	0.87 (0.52–1.47)	0.79 (0.43–1.45)
50+	48 (29.6)	184 (30.5)	0.94 (0.53–1.65)	0.71 (0.35–1.40)
Education Status				
No/informal education	84 (52.0)	332 (55.0)	1.00	1.00
Primary Education	69 (42.6)	215 (5.7)	0.79 (0.55–1.13)	0.66 (0.42–1.05)
Secondary education	9 (5.6)	15 (9.0)	1.54 (0.74–3.30)	0.86 (0.36–2.04)
School child present				
No	17 (10.5)	65 (10.8)	1.00	1.00
Yes	145 (89.5)	537 (89.2)	0.97 (0.55–1.70)	0.90 (0.46–1.73)
Family size				
0–6	97 (60.6)	351 (58.8)	1.00	1.00
> 6	63 (39.4)	246 (41.2)	1.08 (0.76–1.54)	1.06 (0.71–1.59)
Gender				
Male	103 (63.6)	381 (63.0)	1.00	1.00
Female	59 (36.4)	221 (37.0)	1.01 (0.71–1.45)	1.05 (0.67–1.66)
Exposure to communication				
Advised by HEW				
No	20 (12.3)	47 (7.8)	1.00	1.00
Yes	142 (87.7)	555 (92.2)	1.66 (0.95–2.90)	0.60 (0.35–1.05)
Participated in CLTSH				
No	119(73.5)	450 (74.8)	1.00	1.00
Yes	43 (26.5)	152 (25.2)	0.94 (0.63–1.39)	1.02 (0.64–1.62)
Latrine quality factors				
Clean latrine				
No	38 (23.5)	60 (10.0)	1.00	1.00
Yes	124 (75.5)	542 (90.0)	2.77 (1.76–4.35)	1.69 (1.00–3.00)
Latrine has protected entry				
No	144 (89)	454 (75.4)	1.00	1.00
Yes	18 (11)	148 (24.6)	2.61 (1.54–4.40)	1.94 (1.10–3.48)
Latrine has superstructure				
No	96 (59.3)	222 (37)	1.00	1.00
Yes	66 (40.7)	380 (63.0)	2.48 (1.74–3.54) *	2.26 (1.47–3.48)*

When factors that showed significant association in the univariate logistic regression analysis were entered for multivariate logistic regression analysis, one of the psychological factors (attitude) and the three latrine-quality factors remained significant. Participants who had a positive attitude toward latrines were 7 times more likely to be consistent latrine users (95% CI 4.91–11.30). Participants who owned a clean latrine were 1.69 times more likely to be consistent latrine users compared to those who owned dirty latrines (95% CI 1.00–3.00). Latrines that had a superstructure were 2.3 times more likely to be used consistently compared with latrines that did not have a superstructure (95% CI 1.47–3.48). Latrines that had a protected door were 1.94 times more likely to be consistently used than were latrines with no door (95% CI 1.10–3.48).

## Discussion

This cross-sectional quantitative study sought to identify the psychological predictors of latrine ownership in the rural Becho district of Ethiopia. The study found that 73% of participants owned a latrine, and consistent latrine use among latrine owners was 79%. Among the psychological factors, attitude and norm perception predicted latrine ownership. Having a larger family size, the head of household having a higher level of education, having a child attending school, and having a family member who participated in CLTSH triggering were significantly associated with latrine ownership. The findings demonstrated that having a positive attitude toward latrine, owning a clean latrine, owning a latrine with a protected door and a latrine with a superstructure had a significant positive association with consistent latrine use.

The ownership of any kind of latrine by 73% of the participants in this study shows improvement in the country compared with the level of latrine coverage reported by studies in the past decade. Various levels of latrine utilization with some level of geographical variations were reported in previous Ethiopia studies: 61% in rural Hulet Ejju Enessie Woreda [45]; 62% in Bahir Dar Zuria [46], and 45–50% in Hawzen district [47]. However, only 1.5% having access to an improved latrine shows that access to an improved sanitation remains far below any of the targets [18, 48, 49] which needs urgent attention. This finding was close to the 4% access to improved latrines in rural Ethiopia reported by the 2016 Ethiopia DHS report [13, 45, 46] and the WHO and UNICEF JMP reports [6].

This study found that attitude and the injunctive norm were the predictors of latrine ownership, which showed that the social norm influenced people’s decision to own a latrine [40]. Consistent with this finding, a study in Zambia reported that open defecation was commonly practiced because of its acceptance as a societal norm [50]. Given this finding, we believe that normative and persuasive intervention is appropriate for the current setting [27, 51] . CLTSH is a behavior change approach that uses normative and shocking techniques in rural sanitation interventions [51, 52].This study also found that participation in the CLTSH triggering has a significant positive associated with latrine ownership. The current study was conducted in rural Becho, which is a typical rural setting in Ethiopia. We believe that the suggestion of a normative and persuasive approach can be generalized for most of the rural Ethiopia, where 85% of the population resides. However, the generalizability may be limited in some contexts because of the ethnic diversity of A high level of intention to build a latrine in the coming year was reported by the majority of non-adopter household participants. Other studies reported a lower intention to install latrines. For example, a study conducted in Ghana reported a 30% intention level among those who did not own a latrine [53]. However, in this study, given that almost half of intenders reported having low perceived ability (self-efficacy), there was a smaller chance that their intention could be executed [44]. The large majority of non-owners reported having a latrine in the past, and the reason for stopping use of the latrine was because it had collapsed or was broken. This highlighted that latrine ownership might not be sustained. It might be possible that other non-behavioral contextual factors influenced the adoption of a latrine [25]. We recommend further research to explore this.

This study found that attitude was the most powerful predictor of consistent latrine use. The results of this study also showed that cleanliness was associated with consistent latrine use. Consistent with these findings, studies in Ethiopia and other countries also reported that unclean and poorly maintained latrines created a negative perception about latrines and discouraged people from using them [54–56]. The study results show that latrines with a door and latrines with a superstructure were more likely to be consistently used, which might be related to privacy.

The findings from this study show that access to an improved latrine was very limited. This study also highlighted that non-owners have a high intention to install a latrine. Converting this high intention to action (latrine adoption) and sustaining adopted latrines were identified as challenges in the study area. The study also revealed that the socio-demographic status of participants, such as having a better educational status, having a child attending school, and a larger family size influenced latrine ownership. This study found that factors related to latrine quality were significantly associated with consistent latrine use, and among psychological factors, only attitude has a significant association with consistent latrine use. Therefore, efforts to increase latrine adoption should be based on the understanding of multidimensional influences. Investigating the effectiveness of a normative and persuasive approach to health education is suggested for future research. Before generalizing the findings of this study, one should consider that the study was conducted in a very homogenous population, in which 96.4% were farmers, 99% belonged to the Oromo ethnic group, and 99% were followers of Orthodox Christian religion.

## Strength and limitations

- Using a standardized Likert scale adapted from theoretical behavioral models, this study measured the psychological predictors of latrine ownership and consistent latrine use, which was a constraint in previous studies.
- We involved a large sample size and collected preliminary background information using focus group discussion (FGD) as an input for designing the questionnaire, which was the strength of this study.
- We failed to establish causal relationships between potential predictors and the outcome under investigation because our findings were based on a cross-sectional study design.
- We were unable to show the effect of descriptive norm on latrine ownership.

## Conclusion

The study findings show that attitude about latrines and social norms were the psychological predictors of latrine ownership, and consistent latrine use was associated with attitude, the cleanliness of latrine and its privacy. Hence, sanitation intervention needs to focus on changing societal norms and attitudes and maintaining latrine quality.

## Abbreviations

AOR: Adjusted Odds Ratio
CLTSH: Community Led Total Sanitation and Hygien.
DHS: Demographic Health Survey
FMOH: Federal Ministry of Health
OR: Odds Ratio
RANAS: Risk, Attitude, Norm, Ability, Self-regulation
SPSS: Statistical Package for the Social Sciences
TPB: Theory of Planned Behavior
TRA: Theory of Reasoned Action
TSNB: Theory of Social Norm Behavior
UNICEF: United Nations International Children’s Emergency Fund
WASH: Water, Sanitation and Hygiene
